# *p*-Tolyl 2-*O*-benzoyl-3-*O*-benzyl-4,6-*O*-benzyl­idene-1-thio-α-l-idopyran­oside

**DOI:** 10.1107/S1600536810020970

**Published:** 2010-06-09

**Authors:** Graeme J. Gainsford, Peter C. Tyler, Olga V. Zubkova

**Affiliations:** a Carbohydrate Chemistry Team, Industrial Research Limited, PO Box 31-310, Lower Hutt, New Zealand 5040

## Abstract

The title compound, C_34_H_32_O_6_S, is an *ido*-configured thio­glycoside building block for heparan sulfate fragments. It contains disordered tolyl and O-benzyl groups with occupancy ratios of 0.539 (13):0.461 (13) and 0.613 (13):0.387 (13), respectively, as determined from a weakly diffracting crystal. The fused rings adopt chair conformations with the mol­ecules packing into a three-dimensional network *via* C—H⋯O and three C—H⋯π inter­actions. The former inter­actions, occuring between mol­ecules related by a twofold axis, define an *R*_2_^2^(26) motif.

## Related literature

For the synthesis, see: Barroca & Jacquinet (2000 ▶); Polat & Wong (2007 ▶). For a related structure, see: Zhou *et al.* (2006 ▶). For ring conformations, see: Cremer & Pople (1975 ▶) and for hydrogen-bond motifs, see: Bernstein *et al.* (1995 ▶).
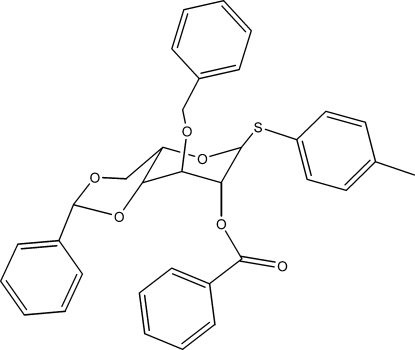


## Experimental

### 

#### Crystal data

C_34_H_32_O_6_S
*M* *_r_* = 568.66
Monoclinic, 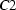

*a* = 19.296 (4) Å
*b* = 8.2060 (16) Å
*c* = 19.045 (4) Å
β = 101.27 (3)°
*V* = 2957.5 (10) Å^3^
*Z* = 4
Cu *K*α radiation
μ = 1.34 mm^−1^
*T* = 123 K
0.60 × 0.11 × 0.11 mm

#### Data collection

Rigaku Spider diffractometer
Absorption correction: multi-scan (*ABSCOR*; Higashi, 1995 ▶) *T*_min_ = 0.754, *T*_max_ = 1.0
10947 measured reflections
4882 independent reflections
2352 reflections with *I* > 2σ(*I*)
*R*_int_ = 0.052

#### Refinement

*R*[*F* ^2^ > 2σ(*F* ^2^)] = 0.068
*wR*(*F* ^2^) = 0.201
*S* = 1.03
4882 reflections
343 parameters
1 restraint
H-atom parameters constrained
Δρ_max_ = 0.28 e Å^−3^
Δρ_min_ = −0.31 e Å^−3^
Absolute structure: Flack (1983 ▶), 1939 Friedel pairs
Flack parameter: 0.01 (4)

### 

Data collection: *CrystalClear* (Rigaku, 2005 ▶); cell refinement: *FSProcess* (Rigaku, 1998 ▶); data reduction: *FSProcess*; program(s) used to solve structure: *SIR92* (Altomare *et al.*, 1993 ▶); program(s) used to refine structure: *SHELXL97* (Sheldrick, 2008 ▶); molecular graphics: *ORTEP* in *WinGX* (Farrugia, 1999 ▶) and Mercury (Macrae *et al.*, 2006 ▶); software used to prepare material for publication: *SHELXL97* and *PLATON* (Spek, 2009 ▶).

## Supplementary Material

Crystal structure: contains datablocks global, I. DOI: 10.1107/S1600536810020970/gk2277sup1.cif

Structure factors: contains datablocks I. DOI: 10.1107/S1600536810020970/gk2277Isup2.hkl

Additional supplementary materials:  crystallographic information; 3D view; checkCIF report

## Figures and Tables

**Table 1:** Hydrogen-bond geometry (Å, °) *Cg*1, *Cg*2 and *Cg*3 are the centroids of the C2,C9–C13, C14*A*–C19*A* and C22–C27 phenyl rings, respectively.

*D*—H⋯*A*	*D*—H	H⋯*A*	*D*⋯*A*	*D*—H⋯*A*
C11—H11⋯O4^i^	0.95	2.50	3.359 (8)	150
C1—H1⋯O2^ii^	1.00	2.62	3.592 (7)	164
C3—H3*A*⋯*Cg*1^ii^	0.99	2.60	3.506 (7)	152
C28*A*—H28*B*⋯*Cg*3^iii^	0.99	2.60	3.572 (11)	165
C31*A*—H31*A*⋯*Cg*2^iii^	0.95	2.87	3.526 (13)	127
C31*B*—H31*B*⋯*Cg*2^iii^	0.93	2.81	3.68 (2)	157

Symmetry codes: (i) 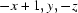; (ii) 
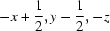
; (iii) 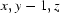.

**Fig. 1.**
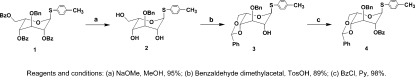
Chemical synthesis steps to the title compound (see text).

**Fig. 2.**
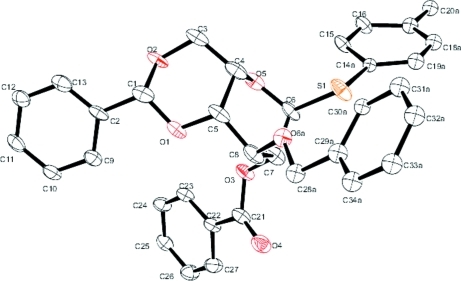
An ORTEP (Farrugia, 1999) view showing the asymmetric unit with 40% probabilility ellipsoids. Only one set (A) of the disordered atoms are shown for clarity.

**Fig. 3.**
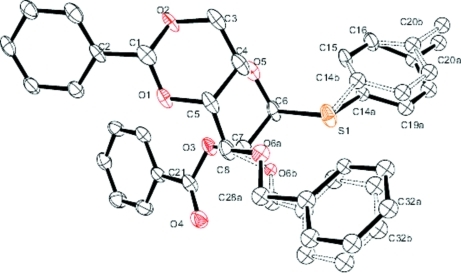
An ORTEP (Farrugia, 1999) view showing the conformational disorder using 40% probabilility ellipsoids. Dotted bonds indicate the minor set (B) atoms; only representattive atoms labels are shown for clarity.

**Fig. 4.**
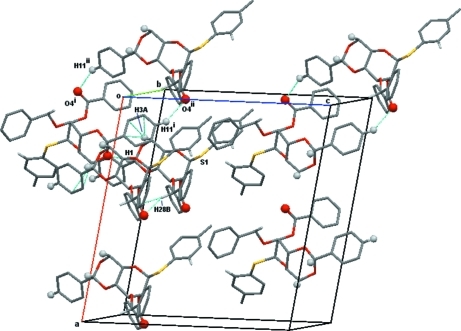
Mercury cell packing view (Macrae et al., 2006) showing most of the C–H···O and C–H···π interactions (dotted lines, Table 1). All contact atoms are in ball mode with other H atoms omitted for clarity. Symmetry operations: (i) 1/2 - x, y - 1/2, -z (ii) x - 1/2, y - 1/2, -z

## supplementary crystallographic information

### Comment

Heparan sulfates (HSs), highly sulfated glycosaminoglycans, have emerged as a novel and exciting class of molecules with a huge variety of critical functions in cell signalling and development. HSs are made up of repeating 1,4-linked disaccharide units. These are composed of a hexuronic acid (*ido-* and *gluco*-configured) and an *N*-acetyl or *N*-sulfoglucosamine which bear one or several *O*-sulfate substituents. *Ido*-configured thioglycoside building blocks 2 and 3 (Figure 1) were prepared to be used in our synthesis of defined fragments of HS.

The title compound (4, Figure 1), C_34_H_32_O_6_S, crystallizes with one independent molecule in the asymmetric unit (Figure 2). For information, its systematic name is benzoic acid 8-benzyloxy-2-phenyl-6-*p*-tolylsulfanyl -hexahydro-pyrano[3,2-*d*][1,3]dioxin-7-yl ester. The phenyl rings (C14–C19 & C29–C34 plus linked atoms C20, O6 & C25) are disordered between two conformations which are labelled a & b respectively (Figure 3) with the final refined occupancies a:b being 0.539 (13):0.461 (13) and 0.613 (10):0.387 (10) respectively. Note that it was not possible to refine two positions at the C15 & C16 sites so these atoms were given unit occupancies.

The determined absolute configuration with C1(*R*), C4(S), C5(*R*), C6(S), C7(*R*) & C8(*R*) confirms the expected stereochemistry and is different from the diacetate derivative (XAZLUG) with configurations *R*,*R*,*S*,*S*,*R*,*S* respectively (Zhou *et al.*, 2006). The fused rings adopt chair configurations: for O1,C1–C5 the puckering amplitude Q is 0.559 (6) Å, θ 166.6 (6)° and φ 243 (3)° while for O5, C4–C8 the corresponding values are 0.525 (6) Å, 13.9 (7)° and 333 (3)° (Cremer & Pople, 1975).

The molecules pack into a three dimensional network using C—H···O and C–H···π interactions (Table 1) with phenyl, tertiary & methylene carbon donor atoms (Figure 2). The C—H···O interactions form a dimeric *R*^2^_2_(26) motif (Bernstein *et al.*, 1995) through O4, between molecules related by the 2-fold rotation axis, and a weaker C(3) link through O1, respectively (Figure 4). The *Cg*1, *Cg*2 & *Cg*3 atom designations in Table 1 are the centroids of phenyl rings (C2,C9–C13), (C14A–C19A) and (C22–C27) respectively. The related diacetate (XUGLAG) packing was reported as two dimensional sheets *via* C–H···O interactions, but these sheets are interconnected *via* at least one C–H···π interaction.

### Experimental

(see Figure 1) *p*-Tolyl 2-*O*-benzoyl-3-*O*-benzyl-4,6-*O*-benzyl idene-1-thio-a-L-idopyranoside (4) was prepared in 4 steps from the known 1,2,4,6-Tetra-*O*-benzoyl-3-*O*-benzyl-β-L-idopyranoside (Barroca & Jacquinet, 2000). The starting tetra-benzoate (7.45 g, 10.85 mmol) was dissolved in (CH_2_Cl_2_, hereafter DCM)(50 ml) and treated with thiocresol (2 g, 16.27 mmol) in the presence of boron trifluoride etherate (0.534 ml, 4.34 mmol) at room temperature for 4 h. The solution was diluted with DCM, washed with water and sat. NaHCO_3_ solution, dried and concentrated. Chromatography (EtOAc: hexanes, 1: 5) furnished the *p*-tolyl-derivative (1, 6.1 g, 8.86 mmol) in 82% yield as a clear syrup. Then Zemplen deacetylation (Polat & Wong, 2007) of the tri-benzoate (1, 6 g, 8.71 mmol) at room temperature afforded a triol (2, 3.1 g, 8.23 mmol) in 95% yield as a syrup. Triol (2, 1 g, 2.66 mmol) was dissolved in dry DMF (15 ml) and treated with benzaldehyde dimethylacetal (1 ml, 6.66 mmol, 2.5 eq.) followed by a catalytic amount (40 mg) of *p*-toluenesulfonic acid. After 1 h at 60°C the solvents were removed *in vacuo* and the residue was purified by flash chromatography on silica gel to give the benzylidene-derivative (3)(1.1 g, 2.37 mmol) in 89% yield as a white foam. The benzylidene-derivative (3, 1 g, 2.15 mmol) was dissolved in a mixture of dry DCM (10 ml) and dry pyridine (10 ml) and cooled to 0°C. Treatment with benzoyl chloride (0.625 ml, 5.38 mmol) at 0°C rising to room temperature for 12 h was followed by an aqueous work-up. The solution was diluted with DCM, washed with water and sat. NaHCO_3_ solution, dried and concentrated. Chromatography (EtOAc: hexanes 1: 4) furnished the benzoate (4, 1.2 g, 2.11 mmol) in 98% yield as a white foam. Compound 4 (100 mg) was dissolved in a hot mixture of EtOAc: hexanes (1:10), and the solution was allowed to cool down slowly. Single crystals were collected and dried *in vacuo*.

^1^H NMR (300 MHz, CDCl_3_) δ 2.29 (s, 3H), 3.91 (ddd, 1H, *J*_3,4_ 2.6 Hz, *J*_2,3_ 2.5 Hz, H-3); 4.12 (dd, 1H, *J*_4,5_ 1.6 Hz, H-4), 4.34 (dd, 1H, *J*_6a,6 b_ 12.3 Hz, H-6a), 4.19 (dd, 1H, H–6 b), 4.51 (ddd, 1H, *J*_5,6a_ 1.5 Hz, *J*_5,6 b_ 2.0 Hz, H-5), 4.71 and 4.95 (2 d, 2H, *J* 11.7 Hz, PhCH_2_), 5.52 (dd, 1H, *J*_2,4_ 1.0 Hz, H-2), 5.56 (s, 1H, PhCH), 5.74 (d, 1H, J_1,2_ 1.3 Hz, H-1), 7.07–8.06 (m, 19H, aromatic). ^13^C NMR (300 MHz, CDCl_3_) δ 21.4, 51.7, 60.9, 68.3, 70.3, 71.5, 72.8, 73.6, 73.7, 77.0, 77.5, 77.9, 86.7, 101.4, 126.8, 127.6, 128.3, 128.4, 128.6, 128.9, 129.2, 129.9, 130.1, 130.6, 130.9, 131.1, 131.4, 133.2, 133.4, 133.9, 137.5, 137.7, 138.3, 166.1. HRMS calcd for C_34_H_32_O_6_S (*M*+Na)^+^ 591.1817, found 591.1824.

The benzoate (4) was converted to the known compound *p*-tolyl 2-*O*-benzoyl-3-*O*-benzyl-1-thio-α-L-idopyranoside (Polat & Wong, 2007). Benzoate (4, 200 mg, 352 µmol) was dissolved in 80% AcOH (10 ml) and stirred at 80°C for 16 h. Concentration and chromatography (EtOAc, hexanes 1: 2) afforded the *p*-tolyl 2-*O*-benzoyl-3-*O*-benzyl-1-thio-α-L-idopyranoside (169 mg, 352 µmol) as a white foam. The ^1^H and ^13^C spectra and mass spectral analyses of this were in accord with literature data (Polat & Wong, 2007).

### Refinement

The methyl H atoms were constrained to an ideal geometry (C—H = 0.98 Å) with *U*_iso_(H) = 1.5*U*_eq_(C), but were allowed to rotate freely about the adjacent C—C bonds. Hydrogen H31B was fixed in a calculated position in the last cycles of refinement. All other H atoms were placed in geometrically idealized positions and constrained to ride on their parent atoms with C—H distances of 0.95 (aromatic), 1.00 (tertiary) or 0.99 (methylene) Å with *U*_iso_(H) = 1.2*U*_eq_(C,*N*). A total of 116 reflections at high theta with negative intensities were clearly outliers (Delta/sigw > 3.5) and were removed from the refinement. One low angle reflection (10,0,0) was also removed as an outlier. A total of 82 reflections out of the 2878 expected within θ 67.7° are therefore not reported. The crystals were poor diffractors but sufficient data was obtained to solve the structure, confirming the absolute configuration.

### Crystal data

**Table:**

C_34_H_32_O_6_S	*F*(000) = 1200
*M**_r_* = 568.66	*D*_x_ = 1.277 Mg m^−^^3^
Monoclinic, *C*2	Cu *K*α radiation, λ = 1.54178 Å
Hall symbol: C 2y	Cell parameters from 1721 reflections
*a* = 19.296 (4) Å	θ = 6.5–66.9°
*b* = 8.2060 (16) Å	µ = 1.34 mm^−^^1^
*c* = 19.045 (4) Å	*T* = 123 K
β = 101.27 (3)°	Needle, colourless
*V* = 2957.5 (10) Å^3^	0.60 × 0.11 × 0.11 mm
*Z* = 4	

### Data collection

**Table:**

Rigaku Spider diffractometer	4882 independent reflections
Radiation source: Rigaku MM007 rotating anode	2352 reflections with *I* > 2σ(*I*)
Rigaku VariMax-HF Confocal Optical System	*R*_int_ = 0.052
Detector resolution: 10 pixels mm^-1^	θ_max_ = 72.2°, θ_min_ = 6.5°
ω–scans	*h* = −22→23
Absorption correction: multi-scan (*ABSCOR*; Higashi, 1995)	*k* = −9→8
*T*_min_ = 0.754, *T*_max_ = 1.0	*l* = −17→23
10947 measured reflections	

### Refinement

**Table:**

Refinement on *F*^2^	Hydrogen site location: inferred from neighbouring sites
Least-squares matrix: full	H-atom parameters constrained
*R*[*F*^2^ > 2σ(*F*^2^)] = 0.068	*w* = 1/[σ^2^(*F*_o_^2^) + (0.0855*P*)^2^] where *P* = (*F*_o_^2^ + 2*F*_c_^2^)/3
*wR*(*F*^2^) = 0.201	(Δ/σ)_max_ = 0.002
*S* = 1.03	Δρ_max_ = 0.28 e Å^−^^3^
4882 reflections	Δρ_min_ = −0.31 e Å^−^^3^
343 parameters	Extinction correction: *SHELXL97* (Sheldrick, 2008), Fc^*^=kFc[1+0.001xFc^2^λ^3^/sin(2θ)]^-1/4^
1 restraint	Extinction coefficient: 0.0019 (2)
Primary atom site location: structure-invariant direct methods	Absolute structure: Flack (1983), 1939 Friedel pairs
Secondary atom site location: difference Fourier map	Flack parameter: 0.01 (4)

### Special details

**Table:**

Geometry. All e.s.d.'s (except the e.s.d. in the dihedral angle between two l.s. planes) are estimated using the full covariance matrix. The cell e.s.d.'s are taken into account individually in the estimation of e.s.d.'s in distances, angles and torsion angles; correlations between e.s.d.'s in cell parameters are only used when they are defined by crystal symmetry. An approximate (isotropic) treatment of cell e.s.d.'s is used for estimating e.s.d.'s involving l.s. planes.
Refinement. Refinement of *F*^2^ against ALL reflections. The weighted *R*-factor *wR* and goodness of fit *S* are based on *F*^2^, conventional *R*-factors *R* are based on *F*, with *F* set to zero for negative *F*^2^. The threshold expression of *F*^2^ > σ(*F*^2^) is used only for calculating *R*-factors(gt) *etc*. and is not relevant to the choice of reflections for refinement. *R*-factors based on *F*^2^ are statistically about twice as large as those based on *F*, and *R*- factors based on ALL data will be even larger.

### Fractional atomic coordinates and isotropic or equivalent isotropic displacement parameters (Å^2^)

**Table:**

	*x*	*y*	*z*	*U*_iso_*/*U*_eq_	Occ. (<1)
S1	0.26771 (9)	0.5118 (2)	0.32352 (10)	0.0904 (6)	
O1	0.3489 (2)	0.4758 (5)	0.0899 (2)	0.0674 (11)	
O2	0.23586 (18)	0.5312 (5)	0.0294 (2)	0.0685 (11)	
O3	0.3923 (2)	0.7274 (5)	0.2062 (2)	0.0671 (11)	
O4	0.5075 (2)	0.7017 (6)	0.2613 (2)	0.0898 (15)	
O5	0.2527 (2)	0.6221 (5)	0.1852 (3)	0.0702 (12)	
C1	0.2993 (3)	0.4463 (7)	0.0262 (4)	0.0712 (18)	
H1	0.2893	0.3269	0.0214	0.085*	
C2	0.3277 (3)	0.5043 (7)	−0.0372 (4)	0.0635 (16)	
C3	0.2032 (3)	0.4603 (8)	0.0837 (4)	0.0777 (19)	
H3A	0.1890	0.3471	0.0698	0.093*	
H3B	0.1599	0.5225	0.0867	0.093*	
C4	0.2516 (3)	0.4592 (7)	0.1565 (4)	0.0746 (19)	
H4	0.2321	0.3829	0.1887	0.090*	
C5	0.3255 (3)	0.4017 (8)	0.1499 (4)	0.0713 (18)	
H5	0.3233	0.2813	0.1417	0.086*	
C6	0.2959 (3)	0.6339 (8)	0.2545 (3)	0.0718 (18)	
H6	0.2945	0.7504	0.2697	0.086*	
C7	0.3720 (3)	0.5973 (7)	0.2490 (3)	0.0703 (18)	
H7	0.4023	0.6025	0.2980	0.084*	
C9	0.3943 (3)	0.5753 (7)	−0.0304 (3)	0.0655 (17)	
H9	0.4232	0.5885	0.0157	0.085*	
C8	0.3821 (3)	0.4344 (7)	0.2166 (4)	0.0738 (19)	
H8	0.4306	0.4243	0.2058	0.089*	
C10	0.4185 (3)	0.6267 (8)	−0.0907 (4)	0.0767 (19)	
H10	0.4640	0.6747	−0.0860	0.092*	
C11	0.3760 (3)	0.6076 (8)	−0.1576 (4)	0.0763 (19)	
H11	0.3926	0.6419	−0.1991	0.092*	
C12	0.3099 (3)	0.5392 (8)	−0.1645 (4)	0.0781 (19)	
H12	0.2807	0.5284	−0.2106	0.094*	
C13	0.2858 (3)	0.4864 (8)	−0.1051 (4)	0.0791 (19)	
H13	0.2404	0.4374	−0.1104	0.095*	
O6A	0.3646 (5)	0.2944 (12)	0.2544 (6)	0.064 (3)*	0.59 (2)
C14A	0.2117 (6)	0.6505 (15)	0.3596 (6)	0.050 (3)*	0.539 (13)
C15	0.1681 (3)	0.7644 (8)	0.3084 (3)	0.0723 (17)	
H15	0.1718	0.7678	0.2594	0.087*	
C16	0.1223 (3)	0.8646 (9)	0.3351 (3)	0.0769 (19)	
H16	0.0924	0.9363	0.3036	0.092*	
C17A	0.1189 (7)	0.8623 (17)	0.4122 (8)	0.0657 (13)*	0.539 (13)
C18A	0.1578 (7)	0.7442 (16)	0.4544 (7)	0.0657 (13)*	0.539 (13)
H18A	0.1529	0.7357	0.5029	0.079*	0.539 (13)
C19A	0.2027 (7)	0.6396 (17)	0.4305 (7)	0.0657 (13)*	0.539 (13)
H19A	0.2277	0.5598	0.4617	0.079*	0.539 (13)
C20A	0.0731 (7)	0.9795 (17)	0.4412 (6)	0.0657 (13)*	0.539 (13)
H20A	0.0361	0.9199	0.4590	0.099*	0.539 (13)
H20B	0.0514	1.0546	0.4032	0.099*	0.539 (13)
H20C	0.1017	1.0414	0.4805	0.099*	0.539 (13)
C21	0.4617 (3)	0.7713 (8)	0.2173 (4)	0.0713 (18)	
C22	0.4727 (3)	0.9110 (7)	0.1729 (3)	0.0613 (16)	
C23	0.4227 (3)	0.9586 (7)	0.1144 (3)	0.0620 (16)	
H23	0.3804	0.8976	0.1009	0.074*	
C24	0.4339 (3)	1.0941 (8)	0.0755 (3)	0.0680 (17)	
H24	0.3990	1.1262	0.0356	0.082*	
C25	0.4956 (3)	1.1845 (9)	0.0941 (3)	0.0729 (17)	
H25	0.5029	1.2786	0.0674	0.088*	
C26	0.5459 (4)	1.1357 (8)	0.1516 (4)	0.081 (2)	
H26	0.5883	1.1966	0.1646	0.097*	
C27	0.5357 (3)	1.0011 (9)	0.1903 (3)	0.0754 (17)	
H27	0.5715	0.9678	0.2294	0.091*	
C28A	0.4234 (5)	0.1966 (16)	0.2865 (6)	0.0727 (11)*	0.613 (10)
H28A	0.4617	0.2669	0.3125	0.087*	0.613 (10)
H28B	0.4418	0.1362	0.2490	0.087*	0.613 (10)
C29A	0.3993 (8)	0.0763 (16)	0.3388 (7)	0.0727 (11)*	0.613 (10)
C30A	0.3302 (5)	0.0774 (15)	0.3568 (6)	0.053 (3)*	0.613 (10)
H30	0.2982	0.1623	0.3386	0.064*	0.613 (10)
C31A	0.3088 (6)	−0.0416 (15)	0.4000 (6)	0.0727 (11)*	0.613 (10)
H31A	0.2633	−0.0393	0.4120	0.087*	0.613 (10)
C32A	0.3585 (6)	−0.1674 (15)	0.4255 (6)	0.0727 (11)*	0.613 (10)
H32A	0.3447	−0.2552	0.4523	0.087*	0.613 (10)
C33A	0.4261 (7)	−0.1638 (16)	0.4120 (6)	0.0727 (11)*	0.613 (10)
H33A	0.4596	−0.2430	0.4332	0.087*	0.613 (10)
C34A	0.4455 (6)	−0.0452 (15)	0.3676 (6)	0.0727 (11)*	0.613 (10)
H34A	0.4916	−0.0478	0.3568	0.087*	0.613 (10)
O6B	0.3821 (6)	0.3383 (16)	0.2850 (8)	0.054 (5)*	0.41 (2)
C14B	0.1955 (9)	0.628 (2)	0.3382 (9)	0.0657 (13)*	0.461 (13)
C17B	0.1005 (8)	0.829 (2)	0.3942 (9)	0.0657 (13)*	0.461 (13)
C18B	0.1331 (8)	0.7014 (19)	0.4340 (8)	0.0657 (13)*	0.461 (13)
H18B	0.1217	0.6782	0.4793	0.079*	0.461 (13)
C19B	0.1832 (8)	0.6041 (19)	0.4088 (8)	0.0657 (13)*	0.461 (13)
H19B	0.2088	0.5229	0.4388	0.079*	0.461 (13)
C20B	0.0442 (8)	0.9276 (19)	0.4240 (7)	0.0657 (13)*	0.461 (13)
H20D	−0.0010	0.8696	0.4136	0.099*	0.461 (13)
H20E	0.0387	1.0353	0.4013	0.099*	0.461 (13)
H20F	0.0594	0.9404	0.4759	0.099*	0.461 (13)
C28B	0.4256 (9)	0.274 (2)	0.3140 (10)	0.0727 (11)*	0.387 (10)
H28C	0.4506	0.3485	0.3515	0.087*	0.387 (10)
H28D	0.4582	0.2522	0.2809	0.087*	0.387 (10)
C29B	0.4141 (11)	0.121 (3)	0.3485 (12)	0.0727 (11)*	0.387 (10)
C30B	0.3541 (13)	0.079 (3)	0.3547 (12)	0.0727 (11)*	0.387 (10)
H30B	0.3156	0.1366	0.3269	0.087*	0.387 (10)
C31B	0.3372 (9)	−0.049 (3)	0.4002 (10)	0.0727 (11)*	0.387 (10)
H31B	0.2946	−0.0856	0.4095	0.087*	0.387 (10)
C32B	0.3913 (10)	−0.137 (2)	0.4345 (10)	0.0727 (11)*	0.387 (10)
H32B	0.3831	−0.2210	0.4664	0.087*	0.387 (10)
C33B	0.4565 (10)	−0.109 (2)	0.4248 (9)	0.0727 (11)*	0.387 (10)
H33B	0.4936	−0.1797	0.4468	0.087*	0.387 (10)
C34B	0.4724 (9)	0.024 (2)	0.3821 (8)	0.0727 (11)*	0.387 (10)
H34B	0.5193	0.0462	0.3765	0.087*	0.387 (10)

### Atomic displacement parameters (Å^2^)

**Table:**

	*U*^11^	*U*^22^	*U*^33^	*U*^12^	*U*^13^	*U*^23^
S1	0.0924 (13)	0.0693 (11)	0.1261 (14)	0.0217 (10)	0.0622 (11)	0.0350 (11)
O1	0.061 (2)	0.055 (3)	0.098 (3)	0.001 (2)	0.045 (2)	0.006 (2)
O2	0.050 (2)	0.058 (3)	0.109 (3)	0.006 (2)	0.044 (2)	0.004 (2)
O3	0.059 (3)	0.064 (3)	0.087 (3)	0.003 (2)	0.035 (2)	0.022 (2)
O4	0.069 (3)	0.105 (4)	0.100 (3)	0.020 (3)	0.030 (2)	0.036 (3)
O5	0.069 (3)	0.041 (2)	0.112 (3)	0.009 (2)	0.045 (3)	0.014 (2)
C1	0.063 (4)	0.040 (3)	0.117 (5)	−0.004 (3)	0.033 (4)	−0.007 (3)
C2	0.044 (3)	0.046 (3)	0.109 (5)	0.002 (3)	0.038 (3)	0.009 (4)
C3	0.065 (4)	0.061 (4)	0.120 (6)	−0.004 (3)	0.049 (4)	0.011 (4)
C4	0.069 (4)	0.043 (4)	0.125 (5)	0.003 (3)	0.050 (4)	0.016 (4)
C5	0.064 (4)	0.047 (4)	0.115 (5)	0.008 (3)	0.049 (4)	0.005 (4)
C6	0.076 (4)	0.053 (4)	0.099 (5)	0.010 (3)	0.049 (4)	0.025 (3)
C7	0.067 (4)	0.059 (4)	0.093 (5)	0.010 (3)	0.034 (4)	0.021 (4)
C9	0.051 (4)	0.067 (4)	0.082 (4)	0.010 (3)	0.021 (3)	0.007 (3)
C8	0.075 (4)	0.052 (4)	0.108 (5)	0.017 (3)	0.050 (4)	0.029 (3)
C10	0.054 (4)	0.093 (5)	0.089 (5)	0.000 (4)	0.028 (4)	0.002 (4)
C11	0.065 (4)	0.075 (5)	0.102 (5)	0.002 (3)	0.048 (4)	−0.020 (4)
C12	0.068 (4)	0.078 (5)	0.095 (5)	0.002 (4)	0.031 (4)	−0.031 (4)
C13	0.066 (4)	0.065 (4)	0.114 (6)	−0.001 (3)	0.037 (4)	−0.032 (4)
C15	0.068 (4)	0.076 (5)	0.084 (4)	0.009 (4)	0.040 (3)	0.007 (4)
C16	0.070 (4)	0.086 (5)	0.084 (5)	0.013 (4)	0.039 (4)	0.020 (4)
C21	0.054 (4)	0.071 (4)	0.097 (5)	0.007 (3)	0.034 (4)	0.008 (4)
C22	0.053 (4)	0.058 (4)	0.079 (4)	0.006 (3)	0.027 (3)	0.018 (3)
C23	0.052 (3)	0.051 (4)	0.086 (4)	0.003 (3)	0.022 (3)	0.009 (3)
C24	0.053 (4)	0.066 (4)	0.087 (4)	0.012 (3)	0.016 (3)	0.014 (4)
C25	0.067 (4)	0.074 (5)	0.086 (5)	0.000 (4)	0.036 (4)	0.010 (4)
C26	0.071 (4)	0.070 (5)	0.103 (5)	−0.021 (4)	0.021 (4)	0.006 (4)
C27	0.049 (4)	0.083 (5)	0.094 (5)	−0.005 (4)	0.015 (3)	0.008 (4)

### Geometric parameters (Å, °)

**Table:**

S1—C14B	1.755 (17)	C20A—H20B	0.9800
S1—C14A	1.796 (13)	C20A—H20C	0.9800
S1—C6	1.818 (6)	C21—C22	1.465 (8)
O1—C1	1.412 (6)	C22—C23	1.379 (7)
O1—C5	1.442 (6)	C22—C27	1.406 (7)
O2—C1	1.420 (6)	C23—C24	1.376 (7)
O2—C3	1.435 (6)	C23—H23	0.9500
O3—C21	1.362 (7)	C24—C25	1.388 (8)
O3—C7	1.443 (6)	C24—H24	0.9500
O4—C21	1.232 (7)	C25—C26	1.375 (8)
O5—C6	1.421 (6)	C25—H25	0.9500
O5—C4	1.443 (7)	C26—C27	1.363 (8)
C1—C2	1.498 (8)	C26—H26	0.9500
C1—H1	1.0000	C27—H27	0.9500
C2—C13	1.392 (7)	C28A—C29A	1.537 (17)
C2—C9	1.394 (7)	C28A—H28A	0.9900
C3—C4	1.513 (8)	C28A—H28B	0.9900
C3—H3A	0.9900	C29A—C34A	1.378 (15)
C3—H3B	0.9900	C29A—C30A	1.441 (17)
C4—C5	1.530 (7)	C30A—C31A	1.391 (15)
C4—H4	1.0000	C30A—H30	0.9500
C5—C8	1.529 (8)	C31A—C32A	1.428 (16)
C5—H5	1.0000	C31A—H31A	0.9500
C6—C7	1.523 (7)	C32A—C33A	1.378 (14)
C6—H6	1.0000	C32A—H32A	0.9500
C7—C8	1.501 (8)	C33A—C34A	1.388 (15)
C7—H7	1.0000	C33A—H33A	0.9500
C9—C10	1.387 (7)	C34A—H34A	0.9500
C9—H9	0.9500	O6B—C28B	1.050 (19)
C8—O6A	1.430 (8)	C14B—C19B	1.42 (2)
C8—O6B	1.522 (12)	C17B—C18B	1.371 (19)
C8—H8	1.0000	C17B—C20B	1.547 (19)
C10—C11	1.384 (8)	C18B—C19B	1.409 (19)
C10—H10	0.9500	C18B—H18B	0.9500
C11—C12	1.377 (8)	C19B—H19B	0.9500
C11—H11	0.9500	C20B—H20D	0.9800
C12—C13	1.374 (8)	C20B—H20E	0.9800
C12—H12	0.9500	C20B—H20F	0.9800
C13—H13	0.9500	C28B—C29B	1.46 (3)
O6A—C28A	1.426 (12)	C28B—H28C	0.9900
C14A—C19A	1.397 (16)	C28B—H28D	0.9900
C14A—C15	1.488 (13)	C29B—C30B	1.24 (3)
C15—C16	1.375 (7)	C29B—C34B	1.43 (2)
C15—H15	0.9500	C30B—C31B	1.44 (3)
C16—C17A	1.484 (15)	C30B—H30B	0.9500
C16—H16	0.9500	C31B—C32B	1.33 (2)
C17A—C18A	1.383 (16)	C31B—H31B	0.922 (19)
C17A—C20A	1.483 (17)	C32B—C33B	1.33 (2)
C18A—C19A	1.359 (15)	C32B—H32B	0.9500
C18A—H18A	0.9500	C33B—C34B	1.43 (2)
C19A—H19A	0.9500	C33B—H33B	0.9500
C20A—H20A	0.9800	C34B—H34B	0.9500
			
C14B—S1—C6	100.0 (6)	C18A—C19A—H19A	120.3
C14A—S1—C6	102.4 (4)	C14A—C19A—H19A	120.3
C1—O1—C5	110.1 (5)	O4—C21—O3	122.4 (6)
C1—O2—C3	109.6 (4)	O4—C21—C22	126.1 (6)
C21—O3—C7	118.3 (5)	O3—C21—C22	111.4 (6)
C6—O5—C4	112.2 (5)	C23—C22—C27	118.5 (6)
O1—C1—O2	109.0 (5)	C23—C22—C21	122.1 (6)
O1—C1—C2	110.2 (5)	C27—C22—C21	119.4 (6)
O2—C1—C2	109.4 (5)	C24—C23—C22	120.3 (6)
O1—C1—H1	109.4	C24—C23—H23	119.8
O2—C1—H1	109.4	C22—C23—H23	119.8
C2—C1—H1	109.4	C23—C24—C25	120.8 (6)
C13—C2—C9	119.2 (6)	C23—C24—H24	119.6
C13—C2—C1	118.6 (6)	C25—C24—H24	119.6
C9—C2—C1	122.2 (6)	C26—C25—C24	118.9 (6)
O2—C3—C4	112.5 (5)	C26—C25—H25	120.5
O2—C3—H3A	109.1	C24—C25—H25	120.5
C4—C3—H3A	109.1	C27—C26—C25	120.9 (6)
O2—C3—H3B	109.1	C27—C26—H26	119.6
C4—C3—H3B	109.1	C25—C26—H26	119.6
H3A—C3—H3B	107.8	C26—C27—C22	120.5 (6)
O5—C4—C3	107.6 (5)	C26—C27—H27	119.8
O5—C4—C5	111.8 (5)	C22—C27—H27	119.8
C3—C4—C5	110.2 (6)	O6A—C28A—C29A	108.9 (9)
O5—C4—H4	109.1	O6A—C28A—H28A	109.9
C3—C4—H4	109.1	C29A—C28A—H28A	109.9
C5—C4—H4	109.1	O6A—C28A—H28B	109.9
O1—C5—C8	107.6 (5)	C29A—C28A—H28B	109.9
O1—C5—C4	112.1 (5)	H28A—C28A—H28B	108.3
C8—C5—C4	113.7 (6)	C34A—C29A—C30A	117.8 (12)
O1—C5—H5	107.7	C34A—C29A—C28A	118.0 (12)
C8—C5—H5	107.7	C30A—C29A—C28A	124.1 (11)
C4—C5—H5	107.7	C31A—C30A—C29A	122.2 (10)
O5—C6—C7	108.7 (5)	C31A—C30A—H30	118.9
O5—C6—S1	115.5 (4)	C29A—C30A—H30	118.9
C7—C6—S1	111.7 (4)	C30A—C31A—C32A	116.8 (10)
O5—C6—H6	106.8	C30A—C31A—H31A	121.6
C7—C6—H6	106.8	C32A—C31A—H31A	121.6
S1—C6—H6	106.8	C33A—C32A—C31A	121.2 (12)
O3—C7—C8	110.9 (5)	C33A—C32A—H32A	119.4
O3—C7—C6	105.4 (4)	C31A—C32A—H32A	119.4
C8—C7—C6	114.1 (6)	C32A—C33A—C34A	120.5 (11)
O3—C7—H7	108.8	C32A—C33A—H33A	119.8
C8—C7—H7	108.8	C34A—C33A—H33A	119.8
C6—C7—H7	108.8	C29A—C34A—C33A	121.3 (12)
C10—C9—C2	120.2 (6)	C29A—C34A—H34A	119.4
C10—C9—H9	119.9	C33A—C34A—H34A	119.4
C2—C9—H9	119.9	C28B—O6B—C8	125.0 (13)
O6A—C8—C7	116.6 (7)	C19B—C14B—S1	111.1 (12)
C7—C8—O6B	95.1 (7)	C18B—C17B—C20B	118.4 (14)
O6A—C8—C5	94.4 (7)	C17B—C18B—C19B	121.1 (15)
C7—C8—C5	111.7 (5)	C17B—C18B—H18B	119.5
O6B—C8—C5	120.1 (8)	C19B—C18B—H18B	119.5
O6A—C8—H8	111.0	C18B—C19B—C14B	119.7 (14)
C7—C8—H8	111.0	C18B—C19B—H19B	120.1
O6B—C8—H8	106.8	C14B—C19B—H19B	120.1
C5—C8—H8	111.0	C17B—C20B—H20D	109.5
C11—C10—C9	119.6 (6)	C17B—C20B—H20E	109.5
C11—C10—H10	120.2	H20D—C20B—H20E	109.5
C9—C10—H10	120.2	C17B—C20B—H20F	109.5
C12—C11—C10	120.3 (6)	H20D—C20B—H20F	109.5
C12—C11—H11	119.8	H20E—C20B—H20F	109.5
C10—C11—H11	119.8	O6B—C28B—C29B	119.4 (18)
C13—C12—C11	120.4 (6)	O6B—C28B—H28C	107.5
C13—C12—H12	119.8	C29B—C28B—H28C	107.5
C11—C12—H12	119.8	O6B—C28B—H28D	107.5
C12—C13—C2	120.2 (6)	C29B—C28B—H28D	107.5
C12—C13—H13	119.9	H28C—C28B—H28D	107.0
C2—C13—H13	119.9	C30B—C29B—C34B	118 (2)
C28A—O6A—C8	114.9 (8)	C30B—C29B—C28B	121 (2)
C19A—C14A—C15	120.8 (10)	C34B—C29B—C28B	120.6 (18)
C19A—C14A—S1	121.8 (9)	C29B—C30B—C31B	126 (2)
C15—C14A—S1	116.9 (8)	C29B—C30B—H30B	117.0
C16—C15—C14A	117.1 (7)	C31B—C30B—H30B	117.0
C16—C15—H15	121.5	C32B—C31B—C30B	116.3 (19)
C14A—C15—H15	121.5	C32B—C31B—H31B	112 (2)
C15—C16—C17A	121.0 (7)	C30B—C31B—H31B	132 (2)
C15—C16—H16	119.5	C33B—C32B—C31B	121 (2)
C17A—C16—H16	119.5	C33B—C32B—H32B	119.6
C18A—C17A—C20A	122.2 (12)	C31B—C32B—H32B	119.6
C18A—C17A—C16	117.4 (11)	C32B—C33B—C34B	121.7 (17)
C20A—C17A—C16	120.4 (11)	C32B—C33B—H33B	119.1
C19A—C18A—C17A	123.9 (12)	C34B—C33B—H33B	119.1
C19A—C18A—H18A	118.1	C29B—C34B—C33B	116.4 (17)
C17A—C18A—H18A	118.1	C29B—C34B—H34B	121.8
C18A—C19A—C14A	119.4 (12)	C33B—C34B—H34B	121.8
			
C5—O1—C1—O2	67.1 (5)	C14B—S1—C14A—C15	−47 (3)
C5—O1—C1—C2	−172.8 (5)	C6—S1—C14A—C15	35.7 (9)
C3—O2—C1—O1	−67.6 (5)	C19A—C14A—C15—C16	4.0 (13)
C3—O2—C1—C2	171.8 (5)	S1—C14A—C15—C16	176.5 (6)
O1—C1—C2—C13	−179.6 (5)	C14A—C15—C16—C17A	2.2 (11)
O2—C1—C2—C13	−59.8 (7)	C15—C16—C17A—C18A	−6.7 (14)
O1—C1—C2—C9	0.3 (7)	C15—C16—C17A—C20A	175.5 (8)
O2—C1—C2—C9	120.1 (6)	C20A—C17A—C18A—C19A	−176.9 (11)
C1—O2—C3—C4	56.9 (6)	C16—C17A—C18A—C19A	5.3 (17)
C6—O5—C4—C3	178.9 (4)	C17A—C18A—C19A—C14A	0.8 (18)
C6—O5—C4—C5	−60.0 (6)	C15—C14A—C19A—C18A	−5.6 (16)
O2—C3—C4—O5	77.3 (6)	S1—C14A—C19A—C18A	−177.8 (9)
O2—C3—C4—C5	−44.9 (6)	C7—O3—C21—O4	1.6 (9)
C1—O1—C5—C8	178.7 (4)	C7—O3—C21—C22	−176.5 (5)
C1—O1—C5—C4	−55.5 (6)	O4—C21—C22—C23	165.3 (6)
O5—C4—C5—O1	−75.6 (7)	O3—C21—C22—C23	−16.6 (8)
C3—C4—C5—O1	44.0 (7)	O4—C21—C22—C27	−15.3 (10)
O5—C4—C5—C8	46.8 (7)	O3—C21—C22—C27	162.7 (5)
C3—C4—C5—C8	166.3 (5)	C27—C22—C23—C24	−1.8 (8)
C4—O5—C6—C7	63.8 (6)	C21—C22—C23—C24	177.6 (5)
C4—O5—C6—S1	−62.7 (5)	C22—C23—C24—C25	0.4 (9)
C14B—S1—C6—O5	−75.0 (7)	C23—C24—C25—C26	0.6 (9)
C14A—S1—C6—O5	−91.3 (6)	C24—C25—C26—C27	−0.2 (10)
C14B—S1—C6—C7	160.1 (7)	C25—C26—C27—C22	−1.2 (10)
C14A—S1—C6—C7	143.8 (6)	C23—C22—C27—C26	2.2 (9)
C21—O3—C7—C8	−85.0 (7)	C21—C22—C27—C26	−177.2 (6)
C21—O3—C7—C6	151.1 (5)	C8—O6A—C28A—C29A	167.0 (10)
O5—C6—C7—O3	65.1 (6)	O6A—C28A—C29A—C34A	170.7 (10)
S1—C6—C7—O3	−166.4 (4)	O6A—C28A—C29A—C30A	−6.1 (15)
O5—C6—C7—C8	−56.8 (6)	C34A—C29A—C30A—C31A	−1.9 (15)
S1—C6—C7—C8	71.8 (6)	C28A—C29A—C30A—C31A	174.8 (11)
C13—C2—C9—C10	−0.3 (9)	C29A—C30A—C31A—C32A	−0.6 (16)
C1—C2—C9—C10	179.8 (6)	C30A—C31A—C32A—C33A	4.5 (16)
O3—C7—C8—O6A	179.3 (7)	C31A—C32A—C33A—C34A	−5.9 (17)
C6—C7—C8—O6A	−61.9 (9)	C30A—C29A—C34A—C33A	0.7 (15)
O3—C7—C8—O6B	161.0 (6)	C28A—C29A—C34A—C33A	−176.3 (10)
C6—C7—C8—O6B	−80.2 (7)	C32A—C33A—C34A—C29A	3.2 (17)
O3—C7—C8—C5	−73.7 (7)	O6A—C8—O6B—C28B	103 (3)
C6—C7—C8—C5	45.1 (7)	C7—C8—O6B—C28B	−114 (2)
O1—C5—C8—O6A	−154.0 (5)	C5—C8—O6B—C28B	127 (2)
C4—C5—C8—O6A	81.2 (6)	C14A—S1—C14B—C19B	−54 (3)
O1—C5—C8—C7	85.1 (6)	C6—S1—C14B—C19B	−153.7 (11)
C4—C5—C8—C7	−39.8 (7)	C20B—C17B—C18B—C19B	178.1 (11)
O1—C5—C8—O6B	−165.0 (6)	C17B—C18B—C19B—C14B	−6(2)
C4—C5—C8—O6B	70.2 (9)	S1—C14B—C19B—C18B	172.3 (10)
C2—C9—C10—C11	0.2 (9)	C8—O6B—C28B—C29B	−144.0 (16)
C9—C10—C11—C12	0.5 (9)	O6B—C28B—C29B—C30B	−12 (4)
C10—C11—C12—C13	−1.2 (10)	O6B—C28B—C29B—C34B	174 (2)
C11—C12—C13—C2	1.2 (10)	C34B—C29B—C30B—C31B	8(4)
C9—C2—C13—C12	−0.4 (9)	C28B—C29B—C30B—C31B	−165.9 (19)
C1—C2—C13—C12	179.5 (6)	C29B—C30B—C31B—C32B	−5(4)
C7—C8—O6A—C28A	−111.1 (10)	C30B—C31B—C32B—C33B	−2(3)
O6B—C8—O6A—C28A	−68.8 (14)	C31B—C32B—C33B—C34B	5(3)
C5—C8—O6A—C28A	131.9 (11)	C30B—C29B—C34B—C33B	−4(3)
C14B—S1—C14A—C19A	125 (4)	C28B—C29B—C34B—C33B	169.7 (17)
C6—S1—C14A—C19A	−151.8 (9)	C32B—C33B—C34B—C29B	−2(3)

### Hydrogen-bond geometry (Å, °)

**Table:**

*Cg*1, *Cg*2 and *Cg*3 are the centroids of the C2,C9–C13, C14A–C19A and C22-C27 phenyl rings, respectively.

**Table:**

*D*—H···*A*	*D*—H	H···*A*	*D*···*A*	*D*—H···*A*
C11—H11···O4^i^	0.95	2.50	3.359 (8)	150
C1—H1···O2^ii^	1.00	2.62	3.592 (7)	164
C3—H3A···Cg1^ii^	0.99	2.60	3.506 (7)	152
C28A—H28B···Cg3^iii^	0.99	2.60	3.572 (11)	165
C31A—H31A···Cg2^iii^	0.95	2.87	3.526 (13)	127
C31B—H31B···Cg2^iii^	0.93	2.81	3.68 (2)	157

Symmetry codes: (i) −*x*+1, *y*, −*z*; (ii) −*x*+1/2, *y*−1/2, −*z*; (iii) *x*, *y*−1, *z*.
